# A convenient method for simultaneous quantification of multiple phytohormones and metabolites: application in study of rice-bacterium interaction

**DOI:** 10.1186/1746-4811-8-2

**Published:** 2012-01-15

**Authors:** Hongbo Liu, Xianghua Li, Jinghua Xiao, Shiping Wang

**Affiliations:** 1 National Key Laboratory of Crop Genetic Improvement, National Center of Plant Gene Research (Wuhan), Huazhong Agricultural University, Wuhan 430070, China

**Keywords:** Abscisic acid, defense, LC-ESI-MS, indole-3-acetic acid, jasmonic acid, phytoalexin, salicylic acid

## Abstract

**Background:**

Simultaneous analysis of multiple functional-related phytohormones and their metabolites will improve our understanding of interactions among different hormones in the same biologic process.

**Results:**

A method was developed for simultaneous quantification of multiple phytohormones, abscisic acid, indole-3-acetic acid (IAA), jasmonic acid (JA), and salicylic acid, hormone conjugates, IAA-aspartic acid, JA-isoleucine, and methyl JA, and phytoalexins, momilactone A, naringenin, and sakuranetin. This method combines a convenient procedure for preparing filtrated crude extracted samples and a sensitive quantification assay using ultra fast liquid chromatography-electrospray ionization tandem mass spectrometry (UFLC-ESI-MS). With this method, we determined the dynamic profiles of defense-related phytohormones, hormone metabolites, and phytoalexins in the interaction of rice with *Xanthomonas oryzae *pv. *oryzae *(*Xoo*), which causes bacterial blight, one of the most devastating diseases of rice worldwide.

**Conclusion:**

This UFLC-ESI-MS method is convenient, sensitive, reliable, and inexpensive for quantification of multiple phytohormones and metabolites compared to current methods. The results obtained by application of this method in studying rice-bacterial interaction provide a basis for understanding the molecular mechanisms of rice defense responses.

## Background

Phytohormones are essential for the regulation of diverse physiologic processes of plants, including development, growth, reproduction, and responses to biotic and abiotic stresses. Plant-produced hormones include nonpeptide hormones, abscisic acid (ABA), auxin or indole-3-acetic acid (IAA, the major form of auxin in most plants), brassinosteroids, cytokinins, ethylene, gibberellins, jasmonic acid (JA), nitric oxide, salicylic acid (SA), and strigolactones, and peptide hormones [1,2]. The nonpeptide phytohormones are structurally unrelated small molecules. Phytohormones act as signal molecules in biological activities and frequently occur in low concentration. The homeostasis of these hormones is tightly controlled between the biosynthetic and metabolic pathways. The metabolism of nonpeptide phytohormones is generally categorized into three types of reactions: hydroxylation, oxidation, and conjugation [3,4]. For example, hydroxylation of JA results in partial biologically active 12-OH-JA and hydroxylation of ABA generates biologically active 7'-OH ABA, 8'-OH ABA, and 9'-OH ABA [3,5]. Cytokinin can be inactivated by oxidation [6]. The formation of hormone conjugates may generate different forms of active hormones, inactive storage hormones, or intermediates for catabolism, such as the active JA-isoleucine (Ile) and methyl JA (MeJA), the inactive storage IAA-alanine, and the intermediate IAA-aspartic acid (Asp) [7-9]. A tiny or small amount of variation in the concentration of a phytohormone may change physiologic activity, although the roles of these hormones in different biologic processes still remain to be elucidated [10]. Thus, quantification of the concentrations of hormones and hormone metabolites is frequently applied in the study of the molecular regulations of different biologic processes.

Accumulating evidence suggests that multiple phytohormones often mediate the same biologic process by additive, synergistic, or antagonistic actions, whereas each type of hormone has a characteristic biologic effect [2,11,12]. For example, plant-pathogen interactions result in changes in the level of various phytohormones [10,13]. SA, JA, and ethylene are well-known signal molecules in plant immunity. Although auxin has a pivotal function in plant development and growth, this hormone also has a position in plant-pathogen interactions [14]. Auxin makes plants susceptible to some biotrophic and hemibiotrophic pathogens [15-17] but resistant to necrotrophic pathogens [18,19]. The auxin-dependent pathway antagonistically interacts with the SA-dependent pathway in the Arabidopsis-pathogen interaction [20] but shares many commonalities with the JA-dependent pathway [2,14]. ABA signaling in abiotic stress responses has been intensively studied [21]. In addition, this hormone is also a player in host-pathogen interactions [22]. ABA can promote disease in some cases and promote defense response in other cases by antagonistic interaction with SA and JA/ethylene or synergistic interaction with JA [22,23].

Because of the complex crosstalk among different hormone signaling pathways and the multifaceted roles of these signaling molecules in a biologic process, simultaneous quantification of multiple hormones and their metabolites in the same sample will facilitate the understanding of the interactions of different hormones. The interactions of different phytohormones frequently occur in a localized tissue in certain biologic processes [2,11,12], which may limit the quantity of tissue samples. Thus, a highly sensitive analytical method is essential for determining the quantitative variation of hormones in the samples that have low concentrations of hormones. Several reports have been published outlining the simultaneous quantification of multiple hormones. Müller et al. [24] reported a multiplex gas chromatography (GC)-tandem mass spectrometry (MS/MS) approach for simultaneous quantification of acidic phytohormones and related compounds, ABA, IAA, JA, SA, and 12-oxo-phytodienoic acid. However, GC-MS/MS analysis requires a complicated sample preparation procedure including separation, purification, and derivatization. Two groups used high-performance liquid chromatography (HPLC)-electrospray ionization (ESI)-MS/MS system to simultaneously quantify acidic hormones ABA, IAA, JA, and SA [25,26]. Further study reported that using HPLC-ESI-MS/MS allows simultaneous quantification of both acidic and basic hormones, IAA, JA, SA, and zeatin, and related metabolites [27]. Kojima et al. [28] reported simultaneous quantification of different molecular species of ABA, cytokinins, gibberellins, and IAA using an ultra-performance liquid chromatography-ESI-MS/MS technique after purifying the samples by solid-phase extraction and chemical derivatization. Although more small molecules were quantified in this LC-MS method [28], the sample preparation procedure is complicated compared to the one used by Durgbanshi et al. [25], Forcat et al. [26], and Pan et al. [27].

Plants also produce secondary metabolites, known as phytoalexins that serve as antibiotics in response to biotic and abiotic stresses. Phytoalexins contribute to plant basal immunity and accumulate around the infection sites soon after the infection of pathogens [29]. However, the relationship between the pathogen infection-responsive crosstalk of different hormone signaling pathways and the accumulation of phytoalexins is poorly understood.

In this study, we established an ultra fast liquid chromatography (UFLC)-ESI-MS/MS-based technique for quantitative analysis of small molecules including phytohormones, hormone metabolites, and phytoalexins. This technique incorporating a convenient sample preparation procedure is sensitive and reliable for simultaneous quantification of multiple analytes in a small amount of tissue. Using this technique, we analyzed the putative interactions of several phytohormones, hormone metabolites, and phytoalexins in rice-bacterium interactions.

## Results and Discussion

### Optimizing quantification conditions

The mass spectrometer was operated in a Q1 scan mode to obtain the mass spectra (MS1) and in a product ion scan mode to obtain the product ion spectra (MS2) of the [M-H] or [M+H] ions. The most abundant precursor and product ion fragmentation for each analyte were selected to carry out the multiple reaction monitoring (MRM) scans. To produce maximal signal for each precursor-product ion transition, different parameters of collision energy, declustering potential, entrance potential, collision cell exit potential, and nitrogen flow rate were tested. Table 1 summarizes the optimized analyzing conditions for the precursor and characteristic product ion of each analyte.

**Table 1:** Optimized MS/MS conditions for quantifying phytohormones and metabolites

**Analyte**^**a**^	**Scan mode**^**b**^	Precursor ion (m/z) Q1	Product ion (m/z) Q3	**Declustering potential (V)**^**c**^	**Entrance potential (V)**^**c**^	**Collision energy (V)**^**c**^	**Collision cell exit potential (V)**^**c**^
SA	-	136.9	92.9	-47	-6	-22	-3
NAA^a^	-	185	141.0	-25	-3	-12	-7
ABA	-	263	153.0	-60	-5	-15	-8
^2^H_6_ABA^a^	-	269	159.1	-57	-5	-16	-8
JA	-	209	59.0	-60	-6	-24	-8.6
DHJA^a^	-	211	59.0	-61	-9	-25	-8
JA-Ile	-	322.2	130.0	-67	-7.5	-32	-7
MeJA	+	225.2	151.2	52	4	16	8
IAA	-	173.9	129.9	-53	-4	-13	-15
IAA-Asp	-	289	131.9	-66	-4	-25	-6
D_2_-IAA^a^	-	176	131.9	-48	-4	-14	-6
Naringenin	-	271.2	151.0	-76	-10	-25	-8
Sakuranetin	-	285.1	119.0	-85	-10	-44	-5
Momilactone A	+	315.2	271.3	82	4	18	6

^a^Internal standards. ^b^-, negative; +, positive. ^c^V, voltage.

### Quantification using UFLC-ESI-MS/MS

Elutes were monitored by a series of MRM scans with scan mode change 700 ms as settle time and 50 ms as dwell time for each precursor-to-product transition. The analyte and its internal standard co-elute in the same or similar time. Because of their different precursor-to-product ion transitions, we specifically detect analyte and its internal standard by extracted ion current from total ion current that combines positive or negative scans. To ensure that the elute peak was the analyte or internal standard, we quantified the elute peak by MRM-triggered information-dependent acquisition-enhanced product ion analysis. The representative LC chromatograms and MS fragmentation patterns of analytes are presented in Additional file 1, Figures S1 and S2. The retention times of analytes were in a range of 8.28 to 19.40 min (Additonal file, Table S1).

To verify the reproducibility of analytes under optimum UFLC-ESI-MS/MS condition, the retention time and peak areas was investigated by repeated injection (n = 5) of a mixture of the standards at a concentration of 10 ng/ml for each standard. The relative standard deviations (RSDs) of the retention times for all the analytes were in a range of 0.0-0.29% (Table 2). The RSDs of peak areas obtained for all the analytes were in a range of 0.73-4.06% (Table 2).

**Table 2:** Calibration curve and other related data for quantifying phytohormones and metabolites

Analyte	Calibration curve		limit of quantification (ng/ml)	Reproducibility (RSD)	
			
	Standard concentration range (ng/ml)	Correlation coefficient		Retention time (%)	Peak area (%)
IAA	1-40	0.9998	0.05	0	0.73
JA	1-100	0.9992	0.01	0	2.42
JA-Ile	1-100	0.9997	0.01	0.26	2.75
ABA	1-100	0.9983	0.01	0	2.29
SA	1-50	0.9995	0.10	0	3.11
IAA-Asp	1-50	0.9902	0.10	0.29	1.77
MeJA	1-50	0.9932	0.10	0.25	2.56
Momilactone A	5-100	0.9987	0.10	0	3.00
Sakuranetin	5-100	0.9993	0.10	0	2.51
Naringenin	5-100	0.9917	0.10	0	4.06

We used the detection limit (signal/noise) >10 as a threshold for quantification of each analyte. The limit of quantification was 0.01 to 0.1 ng/ml for different analyte (Table 2). A calibration curve generated by a series of fixed amounts of standard was used to determine the amount of each analyte in the sample. The concentration ranges of standards used for generating the calibration curves were 1-100 ng/ml (Table 2). The quantities of the analytes in the samples ranged from 1 ng/g to 2000 ng/g fresh weight tissue. The calibration curves were linear in the concentration ranges of the analytes.

### Comparison of different sample preparation procedures

To determine the efficiency of analyte extraction from tissues, we quantified each analyte by UFLC-ESI-MS/MS after each round of analyte extraction in preliminary experiments. After two rounds of analyte extraction, no target analyte could be detected in the purified samples. This result suggests that two rounds of analyte extraction are enough for extracting all the target analytes from tissues. Thus samples were prepared by two rounds of analyte extraction in the following experiments.

Filtration of crude sample extracts before quantification will prolong the lifetime of column in the LC-SEI-MS/MS system, although phytohormones in crude plant extracts can be quantified [26,27]. To ascertain whether different types of filters used for filtration of crude extracted samples would influence analyte recovery, the sample extraction buffer containing standard phytohormones and metabolites was filtrated with either nylon filter or cellulose filter before quantification. The recovery rates of standard phytohormones and metabolites from nylon filter-filtrated samples were ranged from 90% to 95%, and the recovery rates of these standards from cellulose filter-filtrated samples were ranged from 86% to 94% (Additional file 1, Table S1). Although the two types of filters had no significantly different influence (*P *> 0.05) on analyte recovery, the cellulose filter broke frequently during filtration. Thus nylon filter is more suitable for sample preparation in the present experimental condition and was used in the following sample preparation. Durgbanshi et al. [25] reported simultaneous quantification of ABA, IAA, and JA from filtrated plant samples. However, our sample preparation method allows simultaneous quantification multiple phytohormones (ABA, IAA, JA, and SA), hormone metabolites (IAA-Asp, JA-Ile, and MeJA), and phytoalexins (momilactone A, naringenin, and sakuranetin).

The recovery rates of analytes in rice samples were determined by calculating the recovery of internal standards. The recovery of each internal standard was calculated based on the ratio of peak area that was obtained from the extraction of internal standard added to plant samples and the peak area of the same amounts of internal standards measured directly. The recovery rates of internal standards for ABA, IAA, IAA-Asp, JA, JA-Ile, MeJA, and SA after two rounds of extraction ranged from 90% to 95% in rice samples (Additional file 1, Table S1). Because no commercial internal standards are available for phytoalexins, we can not calculate the recovery rates of phytoalexins in rice samples. However, the recovery rates of phytohormones and their metabolites in rice samples showed no significant difference (*P *> 0.05) from the recovery rates of corresponding standards (Additional file 1, Table S1), we argue that the recovery rates of phytoalexins in rice sample might be approximately larger than 90%. These results suggest that the procedure for sample preparation has a good sample recovery and it can be used for quantification of analytes with different polarity.

The samples for quantifying phytohormones are frequently prepared by solid-phase extraction, which purifies sample using a C-bound 18 silica column on the basis of reversed-phase interaction [16,28,30,31]. To ascertain the reliability and sensitivity of our crude extraction procedure for quantifying analytes, grinded powdery samples collected from the leaves of indica rice variety Minghui 63 (*Oryza sativa *ssp. *indica*) and japonica rice variety Zhonghua 11 (*O. sativa *ssp. *japonica*) were divided into two sets. One set of samples was extracted by crude extraction and the other set by solid-phase extraction. Different phytohormones, ABA, IAA, JA, and SA, in these samples were quantified (Table 3). The quantities of the four hormones in the two sets of samples showed no significant difference from each other (*P *> 0.05). These results suggest that the filtrated-crude extract prepared by our sample preparation procedure can be used for UFLC-ESI-MS/MS analysis.

**Table 3:** Comparison of two sample extraction methods for phytohormone quantification

Analyte	Sample extraction	Zhonghua 11		Minghui 63	
			
		**Concentration (ng/g)**^**a**^	*P*	**Concentration (ng/g)**^**a**^	*P*
ABA	crude extraction	38.53 ± 2.92	0.25	20.35 ± 1.16	0.42
	solid-phase extraction	35.66 ± 0.92		21.10 ± 0.85	
IAA	crude extraction	3.23 ± 0.40	0.55	9.91 ± 0.84	0.09
	solid-phase extraction	3.06 ± 0.16		8.56 ± 0.46	
JA	crude extraction	40.78 ± 1.00	0.18	115.05 ± 9.96	0.32
	solid-phase extraction	43.07 ± 2.02		122.50 ± 4.40	
SA	crude extraction	9221.82 ± 732.65	0.61	3390.37 ± 807.91	0.16
	solid-phase extraction	9512.12 ± 545.10		4317.64 ± 291.95	

^a^Each data point represents mean (3 replicates) ± standard deviation.

### Differential hormone and metabolite profiling between resistant and susceptible reactions

Plants protect themselves from pathogen invasion via a complicated signal transduction network. Some phytohormones and metabolites function either as a positive or a negative regulator or factor in this network. ABA, IAA, JA, and SA are reportedly involved in the interactions of rice and different pathogens, although the details of their interactions remains to be elucidated [16,32-34]. The IAA-Asp, which lacks IAA bioactivity, is an intermediate in an irreversible IAA deactivation pathway [35]. Enhanced rice disease resistance to both bacterial and fungal pathogens is accompanied by increased accumulation of IAA-Asp and a reduced level of IAA [16,17]. JA-Ile and MeJA are bioactive jasmonates involved in biotic challenges [36-38], although their roles in rice disease resistance remain to be determined. Phytoalexins are antimicrobial secondary metabolites [29]. Rice momilactone A (a terpenoid phytoalexin) and sakuranetin (a flavonoid phytoalexin) can enhance rice resistance to fungal pathogen [39,40]. Naringenin is also a flavonoid phytoalexin and it can inhibit the growth of both bacterial and fungal pathogens [41]. Bacterial blight caused by *Xanthomonas oryzae *pv. *oryzae *(*Xoo*) is one of the most devastating diseases of rice worldwide. To study the crosstalk of different phytohormones and the relationship and interactions of phytoalexins and hormone-dependent pathways, the dynamic contents of defense-related hormones, ABA, IAA, JA, and SA, their metabolites, IAA-Asp, JA-Ile, and MeJA, and phytoalexins, momilactone A, naringenin, and sakuranetin, in resistant and susceptible reactions were simultaneously quantified and compared using the established UFLC-ESI-MS/MS technique.

**Figure 1:**
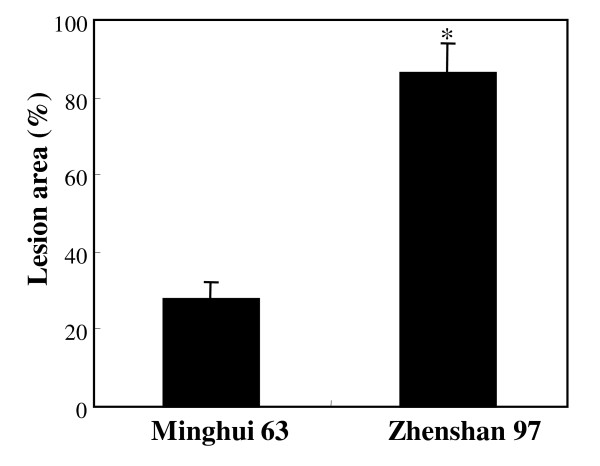
**The response of rice varieties Minghui 63 and Zhenshan 97 to *Xoo *strain PXO61**. Each bar represents mean (5 replicates) ± standard deviation. The asterisk (*) indicates that a significant difference between Minghui 63 and Zhenshan 97 was detected at *P *< 0.01.

Rice variety Minghui 63 carrying major disease resistance genes *Xa3/Xa26 *and *xa25 *against *Xoo *is moderately resistant to *Xoo *strain PXO61, whereas variety Zhenshan 97 is susceptible to *Xoo *[42-45]. The lesion area caused by PXO61 infection was more than 2-fold larger in Zhenshan 97 than in Minghui 63 (Figure 1). The infected leaf tissues were collected for simultaneously quantifying target analytes by UFLC-ESI-MS/MS. The expression patterns of genes known to be involved in hormone and phytoalexin biosynthesis were also analyzed using the same samples and compared to the analyte profiles to examine the reliability of this quantification technique.

#### ABA

**Figure 2:**
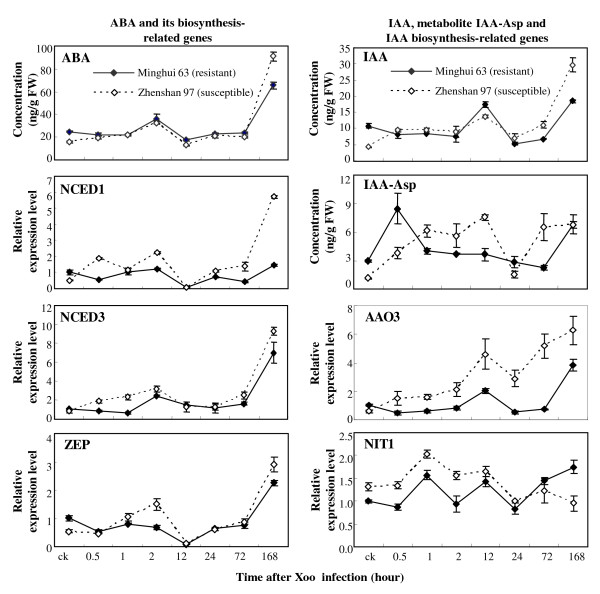
**Infection of *Xoo *strain PXO61 differentially increased the endogenous levels of ABA, IAA, and the metabolite IAA-Asp and the expression of related genes in resistant Minghui 63 and susceptible Zhenshan 97**. Each data point represents mean (3 replicates) ± standard deviation. ck, before *Xoo *infection.

The ABA concentration was 1.6-fold higher in resistant Minghui 63 than in susceptible Zhenshan 97 when without pathogen infection. After inoculation of *Xoo *strain PXO61, the ABA levels in the two rice varieties showed a similar variation pattern in that ABA was significantly increased (*P *< 0.05) at early infection, returned to basal level, and markedly increased again at 7 d after infection (Figure 2). However, the ABA level in Minghui 63 was 27% lower than that in Zhenshan 97 at 7 d after infection. *ZEP *(Rice Genome Annotation Project [http://rice.plantbiology.msu.edu/] locus name: Os04g37619), encoding a zeaxanthin epoxidase, and *NCED*, encoding a 9-*cis*-epoxycarotenoid dioxygenase, are the key genes controlling ABA biosynthesis [46,47]. The three genes, *ZEP*, *NCED1 *(GenBank accession no. AY838897), and *NCED3 *(AY838899), were markedly induced at 7 d after infection with the expression level significantly higher (*P *< 0.05) in Zhenshan 97 than in Minghui 63, which was consistent with the differential accumulation of ABA at 7 d after infection in the two rice varieties (Figure 2). Thus, the expression patterns of these genes further confirmed the reliability of the quantification results.

ABA is involved in plant-pathogen interactions, but its role in these interactions is complex. In plant immunity, ABA functions as a negative signal molecule in some cases but a positive signal molecule in other cases [22,23]. Exogenous application of ABA compromises rice resistance to the fungal pathogen *Magnaporthe oryzae *[34,48]. However, the role of ABA in the interaction of rice and bacterial pathogen *Xoo *is not clear, although protein interaction study around key regulators of biotic response suggests that ABA has an important function in rice resistance to *Xoo *[49]. The present results show that *Xoo*-induced susceptible rice plants accumulated more ABA than the resistant plants. Thus, further study may target the putative negative role of ABA in rice-*Xoo *interaction.

#### IAA

The variation in endogenous IAA levels showed a similar pattern as ABA in the two rice varieties before and after infection with *Xoo*. The concentration of IAA was 2.5-fold higher in resistant Minghui 63 than in susceptible Zhenshan 97 when without pathogen infection. After *Xoo *infection, the IAA levels in the two rice varieties showed a similar variation pattern in that they were significantly increased (*P *< 0.01) at 12 h after infection, returned to basal levels at 24 to 72 h after infection, and markedly increased again at 7 d after infection (Figure 2). However, the IAA level in Minghui 63 was 38% lower than that in Zhenshan 97 at 7 d after infection. Consistent with the IAA level, the IAA-Asp level, the inactive form of IAA, was significantly higher (*P *< 0.01) in Minghui 63 than in Zhenshan 97 before infection and immediately (30 min) after infection (Figure 2). However, the IAA-Asp level was significantly higher (*P *< 0.01) in Zhenshan 97 than in Minghui 63 at 12 h after infection. Indole-3-acetaldehyde oxidase (AAO) and nitrilase (NIT) are two protein families involved in the two tryptophan-dependent pathways for IAA biosynthesis in plants, respectively [7]. The increased expression of rice *AAO3 *(AK065990) and *NIT1 *(AK104033) is associated with increased accumulation of IAA in rice, suggesting that the two genes are most likely involved in IAA synthesis [16,17]. The expression pattern of *AAO3 *was similar to the level of IAA in both varieties before and after *Xoo *infection, suggesting that *AAO3 *may be more closely associated with the local accumulation of IAA after infection than the *NIT1 *gene in the present experimental condition (Figure 2).

Exogenous treatment of rice plants with IAA promotes disease symptoms caused by both bacterial and fungal pathogens [16,17]. Consistent with previous results, the present results also indicate that accumulation of more IAA in susceptible plants is associated with disease. Interestingly, the variation of IAA level in response to *Xoo *infection presented a similar pattern as the variation of ABA level (Figure 2). Although many phytohormones, either as positive or negative players, are involved in plant defense signaling networks [10], no interaction between IAA signaling and ABA signaling has been reported to our knowledge. Thus, it is worth analyzing whether the two hormones function synergistically in the rice-*Xoo *interaction.

#### JA, JA-related compound, and SA

**Figure 3:**
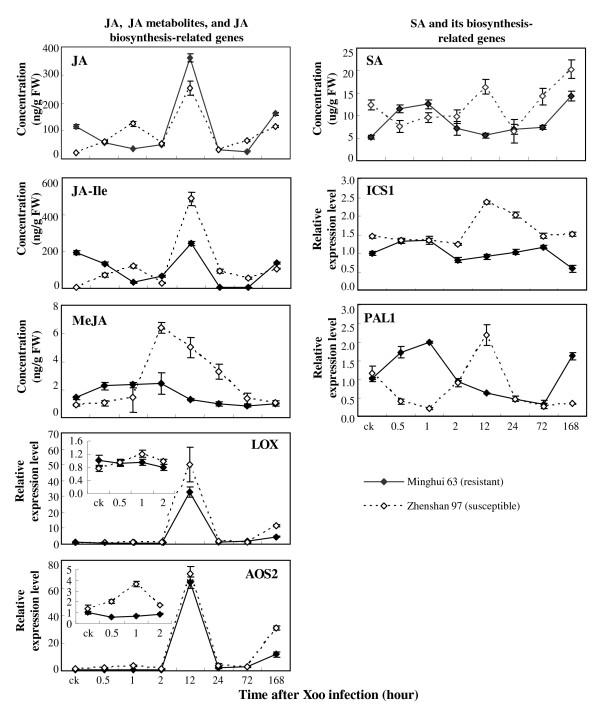
***Xoo *(PXO61) infection influenced endogenous levels of JA, metabolites JA-Ile and MeJA, and SA and the expression of related genes in resistant Minghui 63 and susceptible Zhenshan 97**. Each data point represents mean (3 replicates) ± standard deviation. ck, before *Xoo *infection.

The JA and SA levels showed opposite patterns in the two rice varieties at some time points examined. JA concentration was 4.2-fold higher in resistant Minghui 63 than in susceptible Zhenshan 97, whereas the SA concentration was approximately 58% lower in Minghui 63 than in Zhenshan 97 when without pathogen infection (Figure 3). At early infection (30 min to 1 h), the JA levels were significantly reduced (*P *< 0.01) in Minghui 63 and increased (*P *< 0.01) in Zhenshan 97, but SA levels were significantly increased (*P *< 0.01) in Minghui 63 and reduced in Zhenshan 97 (*P *< 0.05). At 12 h after infection, the JA level was markedly increased, but the SA level returned to the basal level as compared to noninfected plants in Minghui 63, although both JA and SA levels were significantly increased (*P *< 0.05) in Zhenshan 97. At 7 d after infection, the JA level in Minghui 63 was significantly higher (*P *< 0.01) than that in Zhenshan 97, whereas the SA level in Minghui 63 was significantly lower (*P *< 0.05) than that in Zhenshan 97.

JA-Ile, the active form of JA, is a major regulator that controls jasmonate-responsive gene expression and production of secondary metabolites after biotic stress [37]. The variation patterns of JA-Ile levels in response to *Xoo *infection were similar to the JA patterns in the two rice varieties (Figure 3). The exception is that the JA-Ile levels were approximately 2- and 16-fold higher in susceptible Zhenshan 97 than in resistant Minghui 63 at 12 and 24 h after infection, respectively. MeJA is the volatile methylester of JA. It activates JA-dependent defense pathway in plant response to necrotrophic pathogens and insects [36,38]. Minghui 63 had a significantly higher (*P *< 0.01) level of MeJA than Zhenshan 97 at 30 min after infection, but Zhenshan 97 had a significantly higher (*P *< 0.05) level of MeJA than Minghui 63 at 2 to 24 h after infection (Figure 3).

Lipoxygenase (*LOX*; D14000) and allene oxide synthase 2 (*AOS2*; AY062258) regulate JA biosynthesis. Phenylalanine ammonia-lyase 1 (*PAL1*; X16099) is involved in SA biosynthesis by the phenylpropanoid pathway. Isochorismate synthase 1 (*ICS1*; AK120689) is putatively involved in SA biosynthesis in rice by the isochorismate pathway [32]. The expression of *LOX *and *AOS2 *was influenced after infection, which showed similar variation patterns as the variation of JA levels in both Minghui 63 and Zhenshan 97 (Figure 3). *PAL1 *and *ICS1 *also showed expression patterns similar to the variation of SA levels in the two rice varieties (Figure 3). The gene expression results further confirmed the reliability of the quantification data.

Previous reports suggest that multiple JA- and SA-associated pathways may be involved in rice resistance against *Xoo*. For example, overexpressing *MPK6 *or suppressing *WRKY45-1 *or *EDR1 *enhances rice resistance accompanied by activation of both JA and SA signaling [33,50,51]. However, overexpressing *WRKY13 *or suppressing *OsDR10 *or *MPK6 *enhances rice resistance that is accompanied by activation of SA signaling and suppression of JA signaling [32,50,52,53]. In contrast, overexpressing *WRKY45-2 *enhances resistance that is associated with activation of JA signaling but not SA signaling [33]. The opposite JA and SA levels at some time points of the resistance reaction as compared to their levels in susceptible reaction suggest that the JA- and SA-dependent pathways may function antagonistically in rice resistance to *Xoo*. According to this inference and previous reports [32,33,50-53], we argue that the JA-and SA-dependent pathways may have crisscross roles in rice-*Xoo *interactions.

The IAA-dependent pathway may act antagonistically to the SA-dependent defense pathway in Arabidopsis-pathogen interaction; SA-mediated plant immunity accompanies the repression of the auxin signaling in Arabidopsis [20,54]. However, the dynamic profiling of IAA and SA after *Xoo *infection did not show obvious antagonistic interaction (Figures 2 and 3). Previous studies have also revealed that enhancing rice resistance to *Xoo *by suppressing IAA signaling does not require activation of SA signaling [16,17]. These results suggest that in rice-pathogen interaction, IAA signaling and SA signaling may interact in a different way from that in Arabidopsis.

#### Phytoalexins

**Figure 4:**
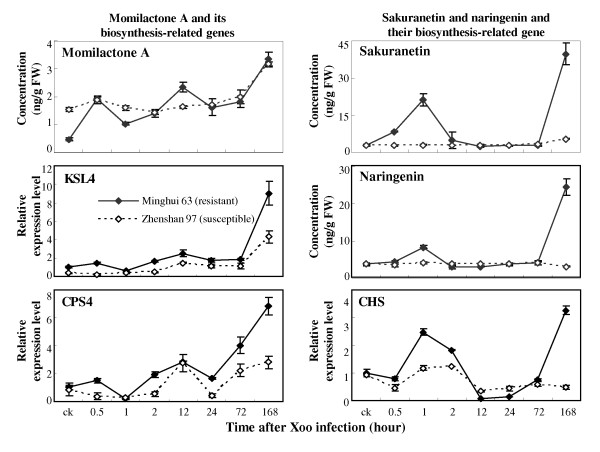
***Xoo *(PXO61) infection induced accumulation of terpenoid phytoalexin momilactone A and flavonoid phytoalexins sakuranetin and naringenin and the expression of related genes in rice**. Each data point represents mean (3 replicates) ± standard deviation. ck, before *Xoo *infection.

We used an external standard method for quantification of phytoalexins as reported previously [55]. The concentration of momilactone A was approximately 69% lower in resistant Minghui 63 than in susceptible Zhenshan 97 when without pathogen infection (Figure 4). However, *Xoo *infection more rapidly increased momilactone A accumulation in Minghui 63 than in Zhenshan 97. Consistent with the accumulation of momilactone A, *Xoo *infection also induced the expression of momilactone A biosynthesis-related genes *KSL4 *(AK119327) and *CPS4 *(AK100631) that encode *syn*-pimaradiene synthase and *syn*-CDP synthase, respectively [56,57]. The levels of sakuranetin and naringenin showed a similar pattern of variation in resistant Minghui 63 after infection (Figure 4), which is consistent with the fact that naringenin is the precursor of sakuranetin [40]. After infection, the sakuranetin and naringenin levels were rapidly increased at 1 h, return to the basal levels at 2 h, and markedly increased again at 7 d in Minghui 63, but no such induction was detected in susceptible Zhenshan 97. *CHS *(X89859) encodes chalcone synthase that catalyzes the first step in the biosynthesis of flavonoids [58,59]. The expression of *CHS *showed a similar variation pattern as the patterns of sakuranetin and naringenin levels in response to *Xoo *infection (Figure 4).

The differentially increased accumulation of sakuranetin and naringenin in the resistance reaction but not in the susceptible reaction suggest that the two flavonoid phytoalexins may be involved in *Xoo *resistance in the present experimental conditions. This inference is also supported by the evidence that naringenin displayed growth inhibition of *Xoo *in culture [41]. The terpenoid phytoalexin momilactone A can inhibit the growth of rice fungal pathogen *M. oryzae *[39,40,55], but its role in rice resistance to *Xoo *is not clear. Although *Xoo *infection increased the accumulation of momilactone A in both resistant and susceptible rice plants, the concentrations of momilactone A in the two rice varieties showed no significant difference (*P *> 0.05) at 7 d after infection. In addition, rice WRKY13-mediated *Xoo *resistance does not accompany increased accumulation of momilactone A [52]. Thus, the role of momilactone A in rice-*Xoo *interaction remains to be elucidated.

The flavonoid phytoalexins are synthesized via a branch, which is different from the branch for SA biosynthesis, in the phenylpropanoid pathway [52]. PAL is an important enzyme upstream of the two branches in this pathway. The *PAL1 *showed a 4.6-fold higher expression level in resistant Minghui 63 than that in susceptible Zhenshan 97, but the SA level in the former was significantly lower (*P *< 0.05) than that in the latter at 7 d after infection (Figure 3). This may be explained by the fact that the phenylpropanoid pathway largely contributes to the biosynthesis of phytoalexins in the resistant reaction (Figure 4).

## Conclusion

In comparison with published methods [24-28], the advantage of the present method is the convenient and inexpensive procedure for preparing filtrated crude extracted samples, which does not require a complicated procedure of sample derivatization or has less damage to the columns of the LC-ESI-MS/MS system. Furthermore, this convenient sample preparation method can be used for simultaneous quantification of multiple phytohormones and metabolites that have different polarity. By simultaneous quantification of multiple hormones and their metabolites and phytoalexins using this method and considering previous reports, we suggest that the JA- and SA-dependent pathways may function antagonistically in rice resistance to *Xoo*; however, the two pathways may have crisscross roles in the rice-*Xoo *interaction. In addition, at late infection, the phenylpropanoid pathway may largely contribute to the biosynthesis of flavonoid phytoalexins that can directly inhibit *Xoo *growth other than the biosynthesis of SA.

## Methods

### Chemicals

The standard IAA, IAA-Asp, JA, MeJA, and SA were purchased from Sigma-Aldrich (St. Louis, MO, USA), and ABA and JA-Ile were from OlChemIm (OlChemIm, Olomouc, Czech Republic). The internal standards were ^2^H_6_ABA (Olchemin) for ABA, 10-dihydro-JA (DHJA; Olchemin) for JA, JA-Ile, and MeJA, D_2_-IAA (Sigma-Aldrich) for IAA and IAA-Asp, and naphthalene acetic acid (NAA; internal standards) for SA. The standard momilactone A was kindly provided by Dr. Morifumi Hasegawa of College of Agriculture, Ibaraki University. The standard naringenin and sakuranetin were purchased from Extrasynthèse (Genay, France).

### Bacterial inoculation

Rice varieties (*Oryza sativa *ssp. *indica*) Minghui 63 and Zhenshan 97 were inoculated with *Xoo *strain PXO61 by leaf-clipping method at booting (panicle development) stage [42]. Disease was scored by measuring the percentage disease area (lesion length/leaf length) at 2 weeks after inoculation. The 2-cm leaf fragments next to bacterial infection sites were collected and stored at -70°C for preparing samples for UFLC-ESI-MS/MS analysis and gene expression analyses.

### Sample preparation

Samples were prepared using a modified crude extraction procedure that was originally reported by Pan et al. [27]. Three replicates of each frozen leaf sample (~100 mg for each replicate) were ground to a fine power in liquid nitrogen using a mortar and pestle. Each sample was weighed into a 1.5-mL tube, mixed with 750 μL cold extraction buffer (methanol:water:acetic acid, 80:19:1, v/v/v) supplemented with internal standards, 10 ng ^2^H_6_ABA, 10 ng DHJA, 5 ng D_2_-IAA, and 3 μg NAA, vigorously shaken on a shaking bed for 16 h at 4°C in dark, and then centrifuged at 13,000 rpm for 15 min at 4°C. The supernatant was carefully transferred to a new 1.5-mL tube and the pellet was remixed with 400 μL extraction buffer, shaken for 4 h at 4°C, and centrifuged. The two supernatants were combined and filtered using a syringe-facilitated 13-mm diameter nylon filter with pore size 0.22 μm (Nylon 66; Jinteng Experiment Equipment Co., Ltd, Tianjing, China). The filtrate was dried by evaporation under the flow of nitrogen gas for approximately 4 h at room temperature, and then dissolved in 200 μL methanol. A aliquot of dissolved sample was further diluted 100 times using methanol for quantification of SA, because rice contains a high level of SA.

To determine whether different types of filters would influence the recovery rates of analytes, each standard (5 ng) was added into a 1.5-mL tube, mixed with the same amount of cold extraction buffer, vigorously shaken on a shaking bed, and centrifuged as described above. The samples were then filtered using a nylon filer or a syringe-facilitated 13-mm diameter cellulose filter with pore size 0.22 μm (MCE; Navigator Lab Instrument Co., Ltd, Tianjing, China). The filtrates were dried and then dissolved in methanol as described above.

For comparison, samples were also prepared using a solid-phase extraction procedure [16]. In brief, ground sample powder was mixed with 2 ml extraction buffer and shaken on a shaking bed for 16 h as for the above-described preparation for crude extraction, and then centrifuged at 3500 *g *for 15 min at 4°C for collecting the supernatant. The supernatant was purified using a C18-SepPak cartridge (Waters Corporation, Milford, MA, USA) by a series of steps. The purified sample was dried by evaporation and then dissolved in 200 μL of methanol as described above for preparation for the crude extraction.

### UFLC-ESI-MS/MS

Liquid chromatography was carried out using a UFLC with an autosampler (Shimadzu Corporation, Kyoto, Japan). A Waters Atlantis T3 (Waters Corporation) column (2.1 × 150 cm, 3 μm) was used at ambient temperature. The injected volume of sample was 10 μL. The elution gradient was carried out with binary solvent system consisting of 0.02% acetic acid in H_2_O (solvent A) and 0.02% acetic acid in MeCN (solvent B) at a constant flow rate of 250 μL/min. A linear gradient profile with the following proportions (v/v) of solvent B was applied: gradient profile 0 to 5 min and 0% of B, 5 to 8 min and 0% to 16% of B, 8 to 20 min and 16% to 100% of B, 20 to 25 min and 100% of B, 25 to 28 min and 100% to 0% of B, and 28 to 32 min with 4 min for re-equilibration and 0% of B.

To diagnose the hormone precursor-to-product ion transitions, mixtures of 150 ng/mL of the standard compounds dissolved in 50% MeCN were directly infused into a hybrid triple quadrupole/linear ion trap mass spectrometer (ABI 4000 Q-Trap, Applied Biosystems, Foster City, CA, USA) outfitted with an electrospray ion source. The analysis parameters were optimized for the production of characteristic precursor-to-product ion transitions in negative or positive ionization modes. ABA, IAA, IAA-Asp, JA, JA-Ile, SA, sakuranetin, naringenin, and their internal standards were scanned in the negative mode, whereas momilactone A and MeJA were analyzed in the positive mode. The mixtures of standard compounds were separated by reversed-phase UFLC and analyzed by ESI-MS/MS in the MRM mode with 50 ms dwell time, 5 ms of pause time between mass ranges, and 700 ms of settle time for switching polarities. The identities of phytohormones and metabolites in the crude plant extracts were confirmed by analysis of product ion fragments obtained by the hybrid triple quadrupole/linear ion trap mass spectrometer, operating in the IDA mode, with a source voltage of 4.5 kV and source temperature of 550. In the ''Enhanced Product Ion" scan mode, precursor ions were fragmented with collision energy +25 kV or -25 kV and products in the range of 50 to 500 m/z were detected.

UFLC-ESI-MS/MS assays were repeated twice biologically, with each repetition having three replicates. Similar results were obtained in repeated experiments; only the result in one repetition was presented.

### Gene expression analyses

Quantitative reverse transcription-polymerase chain reaction (qRT-PCR) was performed as described previously [32]. The gene-specific primers are listed in Additional file 1, Table S2. The expression level of actin gene was first used to standardize the RNA sample for each qRT-PCR. The expression level relative to control was then presented. For each gene, qRT-PCR assays were repeated twice biologically, with each repetition having three replicates. Similar results were obtained in repeated experiments; only the result in one repetition was presented.

### Statistical analysis

The significant differences between control and treatment of the samples were analyzed by the pair-wise *t *test installed in the Microsoft Office Excel program.

## Competing interests

The authors declare that they have no competing interests.

## Authors' contributions

HL performed LC-ESI-MS and gene expression analyses and drafted the manuscript. XL and JX provided molecular analysis support. SW contributed to data interpretation and to writing the manuscript. All authors read and approved the final manuscript.

## Supplementary Material

Additional file 1 **Figure S1 Representative LC chromatograms of analytes and their internal standards**. Figure S2 Representative MS fragmentation patterns of analytes. Table S1 Recovery of analytes in standard-containing samples prepared using different filters and in rice samples. Table S2 Primers used for quantitative reverse-transcription-PCR analysis. Click here for file
