# Supplementary material for: A convenient method for simultaneous quantification of multiple phytohormones and metabolites: application in study of rice-bacterium interaction
Source: Plant Methods. 2012 Jan 15;8:2. doi: 10.1186/1746-4811-8-2 (PMC3274484; doi:10.1186/1746-4811-8-2)
Supplement: Additional file 1 — Figure S1 Representative LC chromatograms of analytes and their internal standards. Figure S2 Representative MS fragmentation patterns of analytes. Table S1 Recovery of analytes in standard-containing samples prepared using different filters and in rice samples. Table S2 Primers used for quantitative reverse-transcription-PCR analysis. [file 1746-4811-8-2-S1.PDF]

A convenient method for simultaneous quantification of multiple phytohormones and metabolites: application in study of rice–bacterium interaction

Hongbo Liu, Xianghua Li, Jinghua Xiao and Shiping Wang

Additional file 1: Supplemental figures and tables

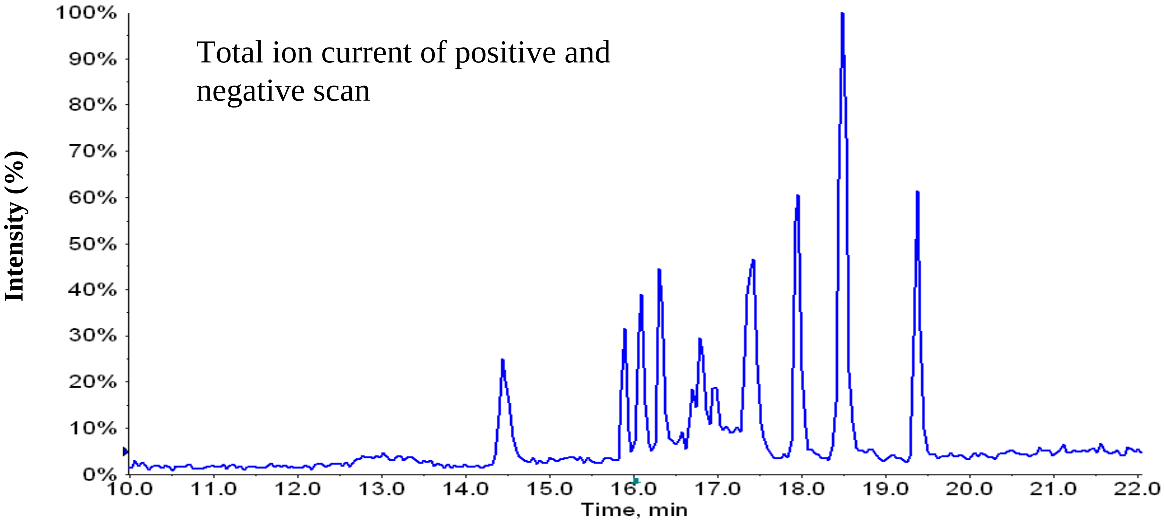

Figure S1. Representative LC chromatograms of analytes and their internal standards.

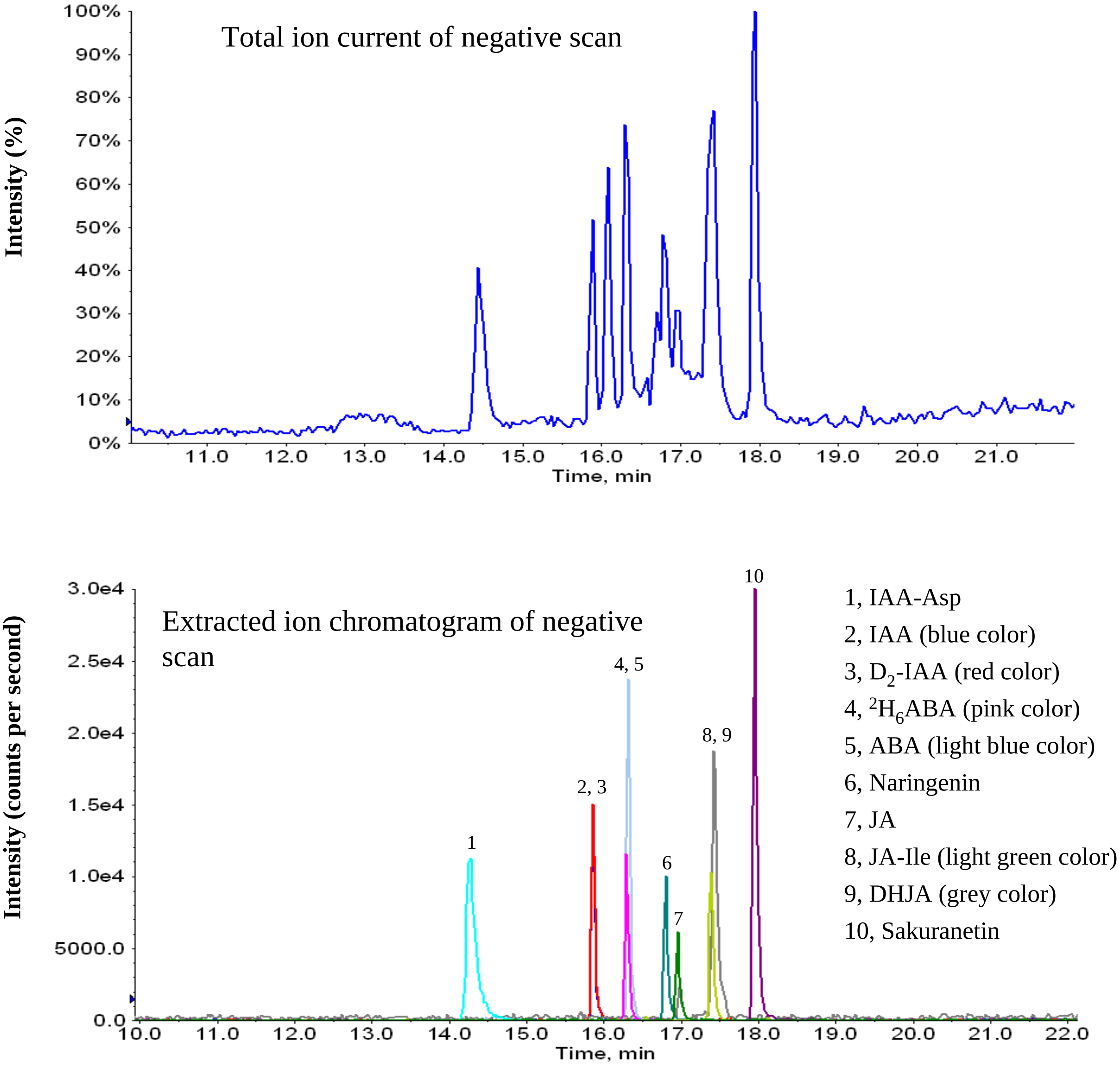

Figure S1 (continued 1). Representative LC chromatograms of analytes and their internal standards.

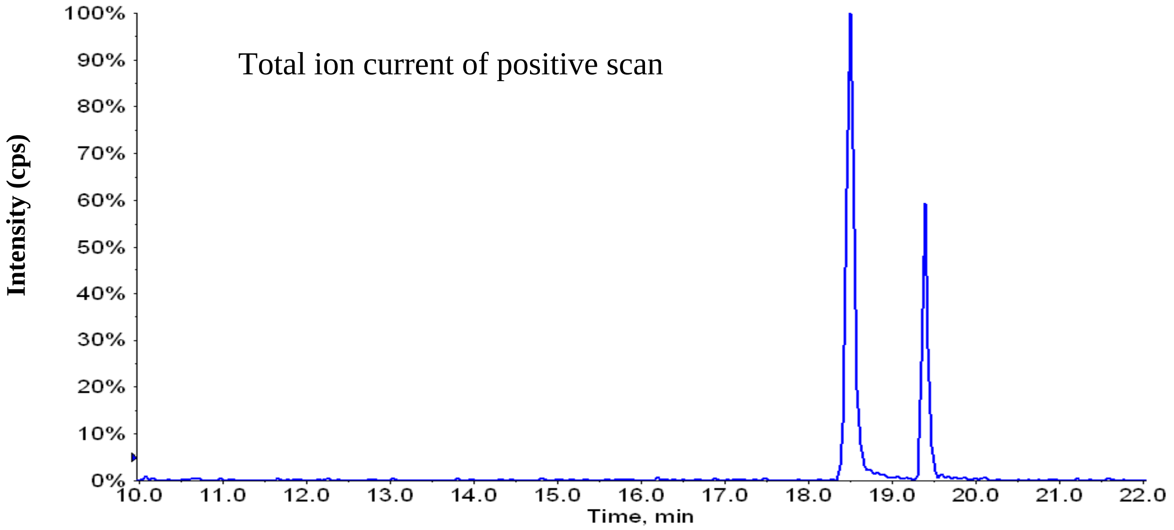

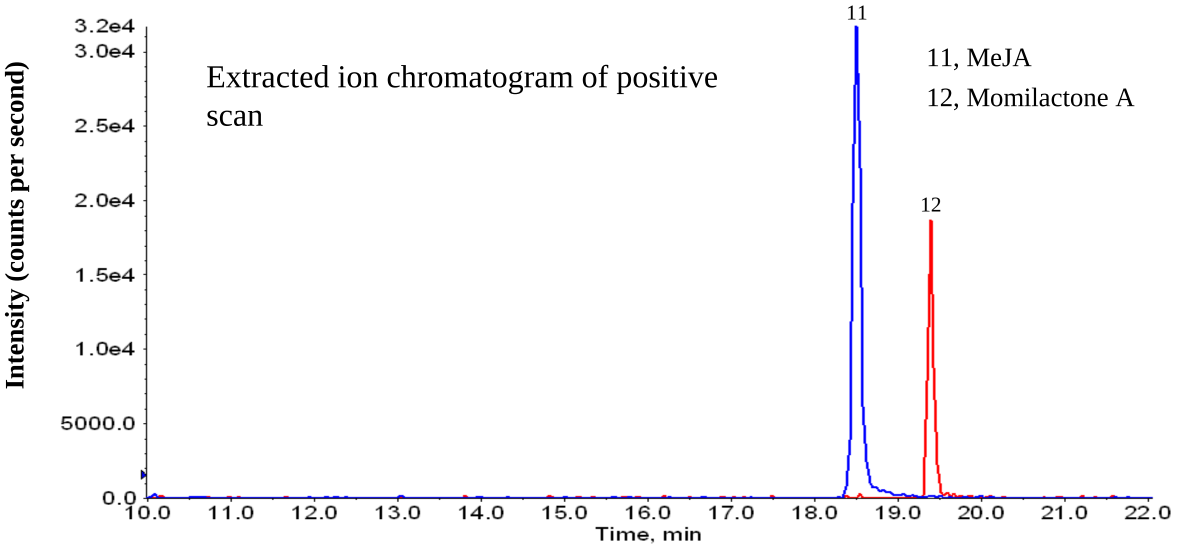

Figure S1 (continued 2). Representative LC chromatograms of analytes and their internal standards.

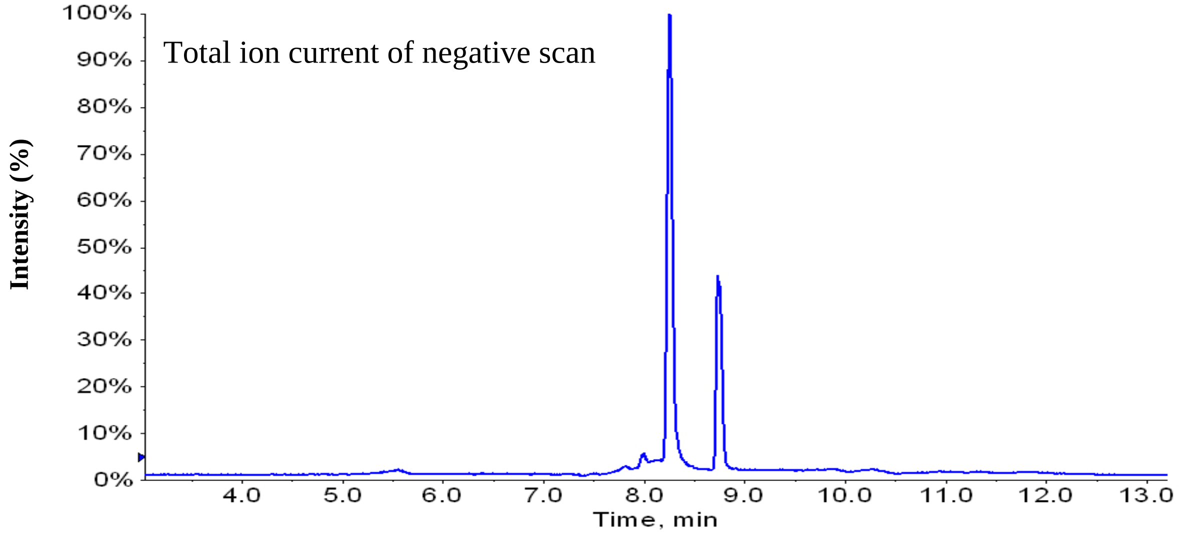

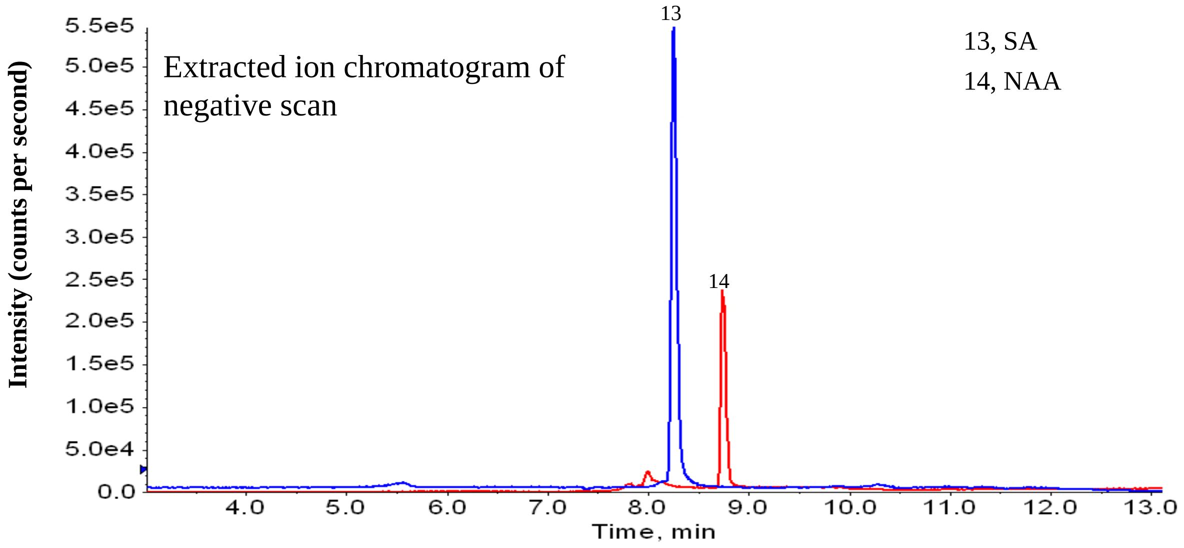

Figure S1 (continued 3). Representative LC chromatograms of analytes and their internal standards.

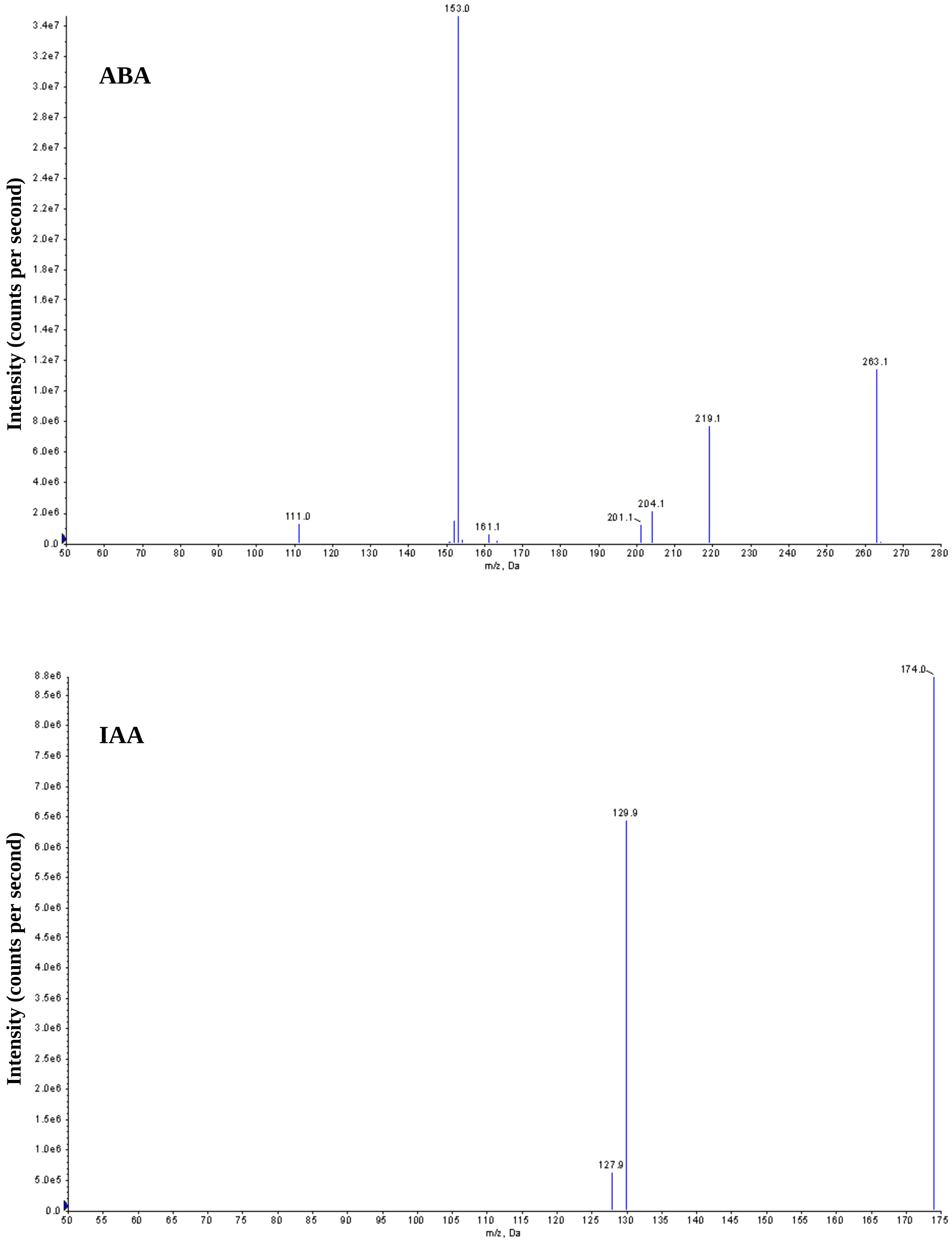

Figure S2. Representative MS fragmentation patterns of analytes.

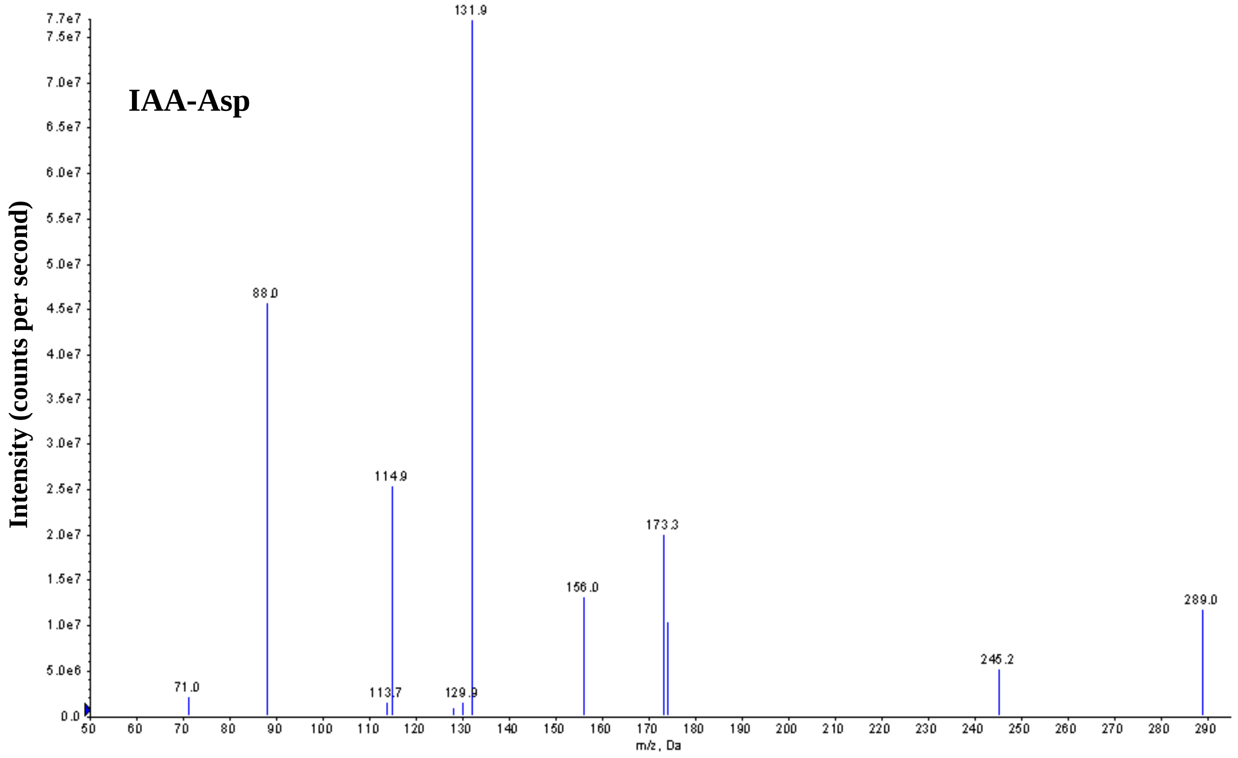

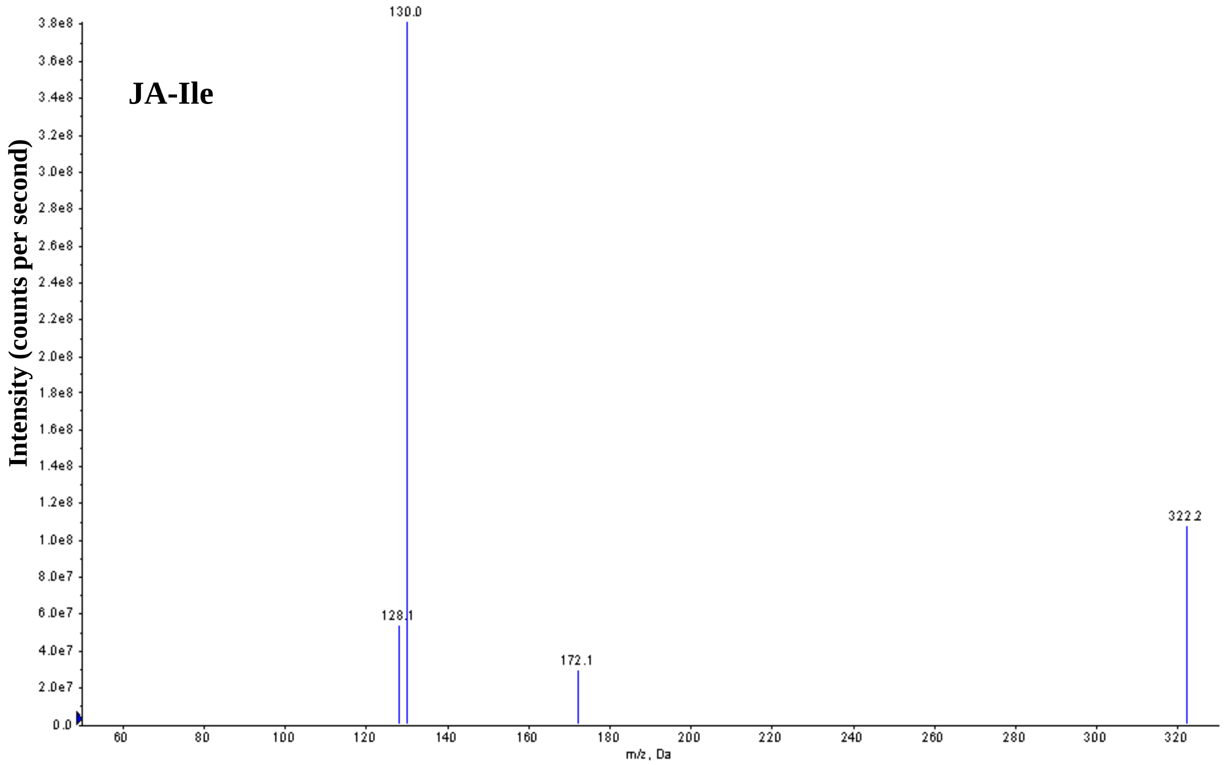

Figure S2 (continued 1). Representative MS fragmentation patterns of analytes.

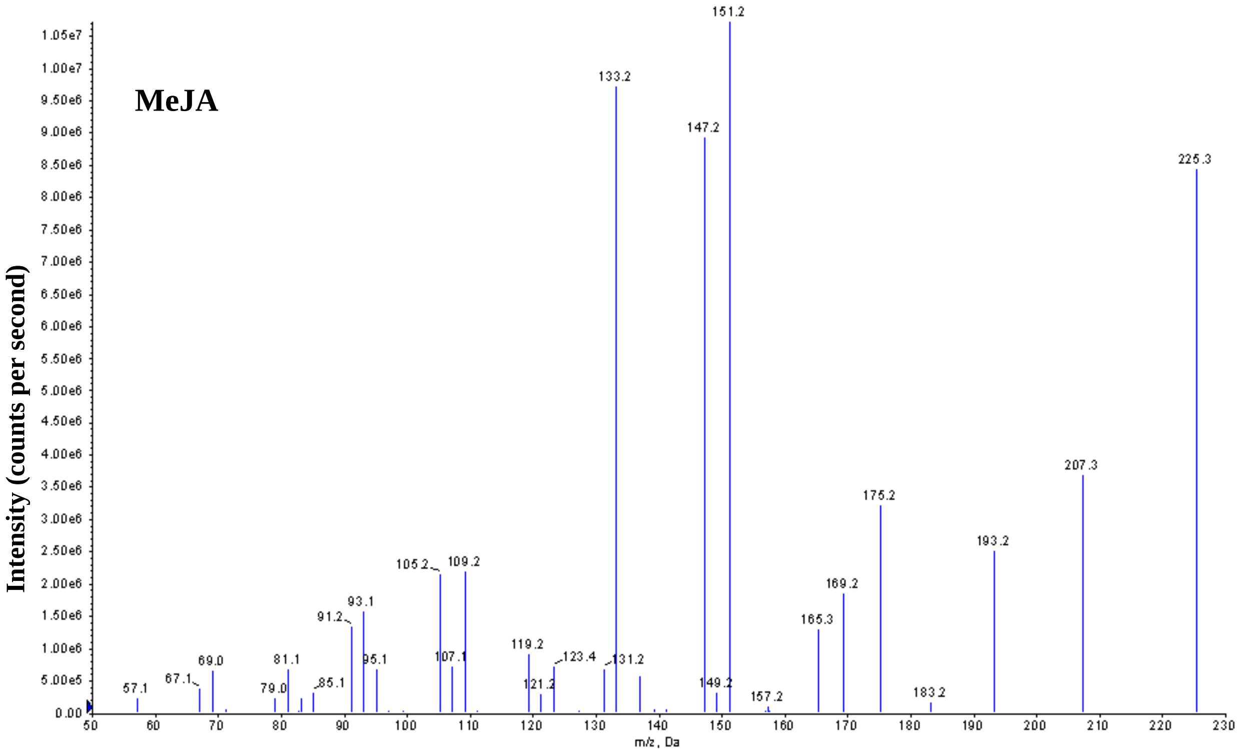

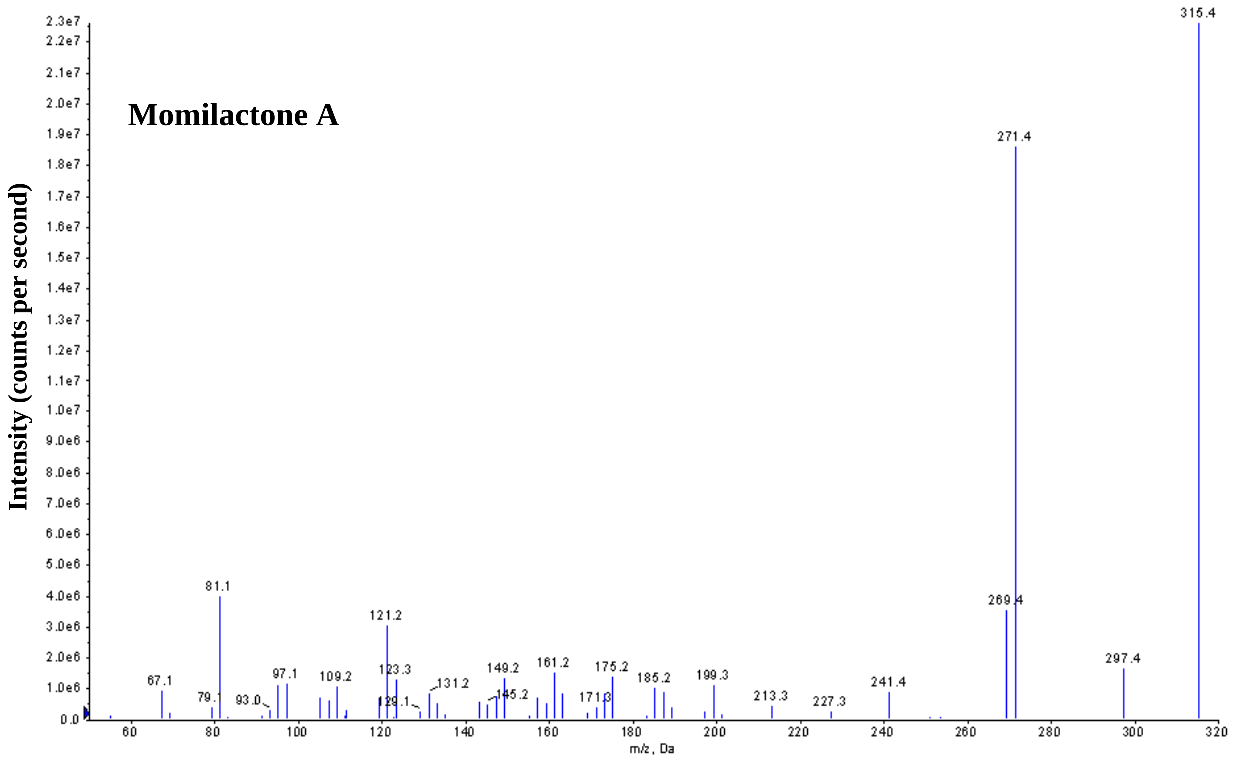

Figure S2 (continued 2). Representative MS fragmentation patterns of analytes.

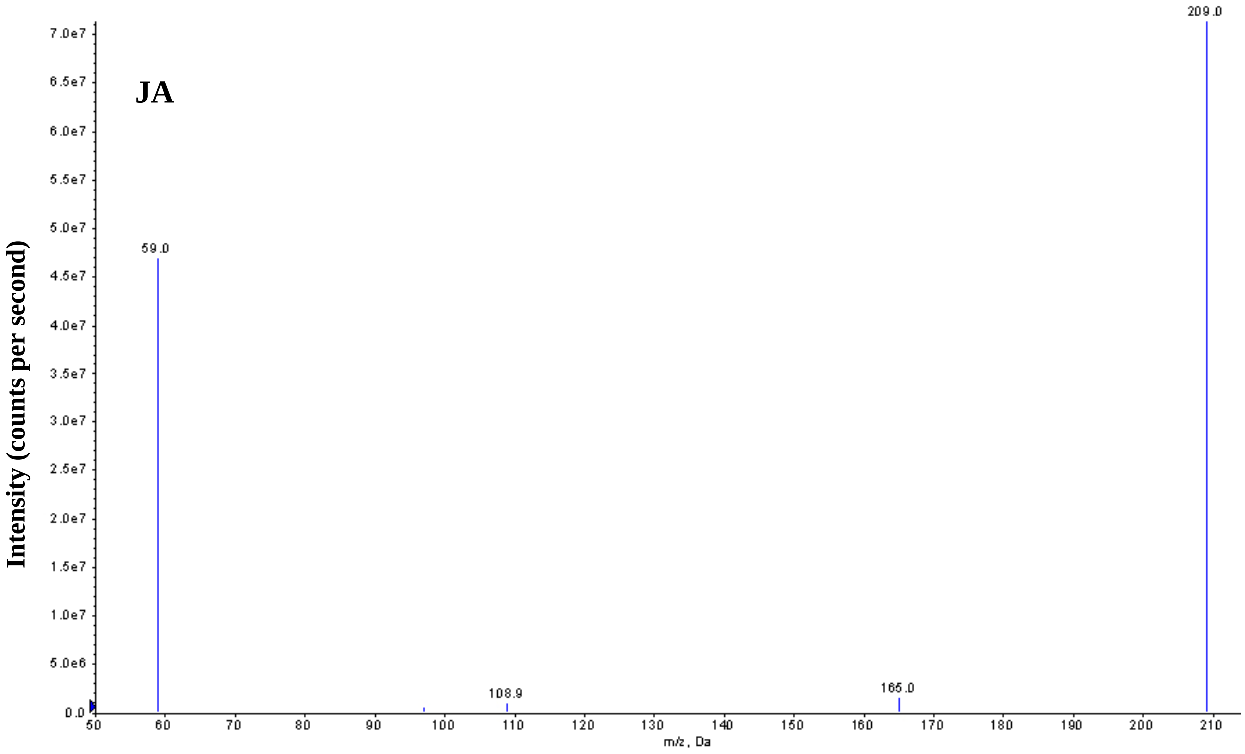

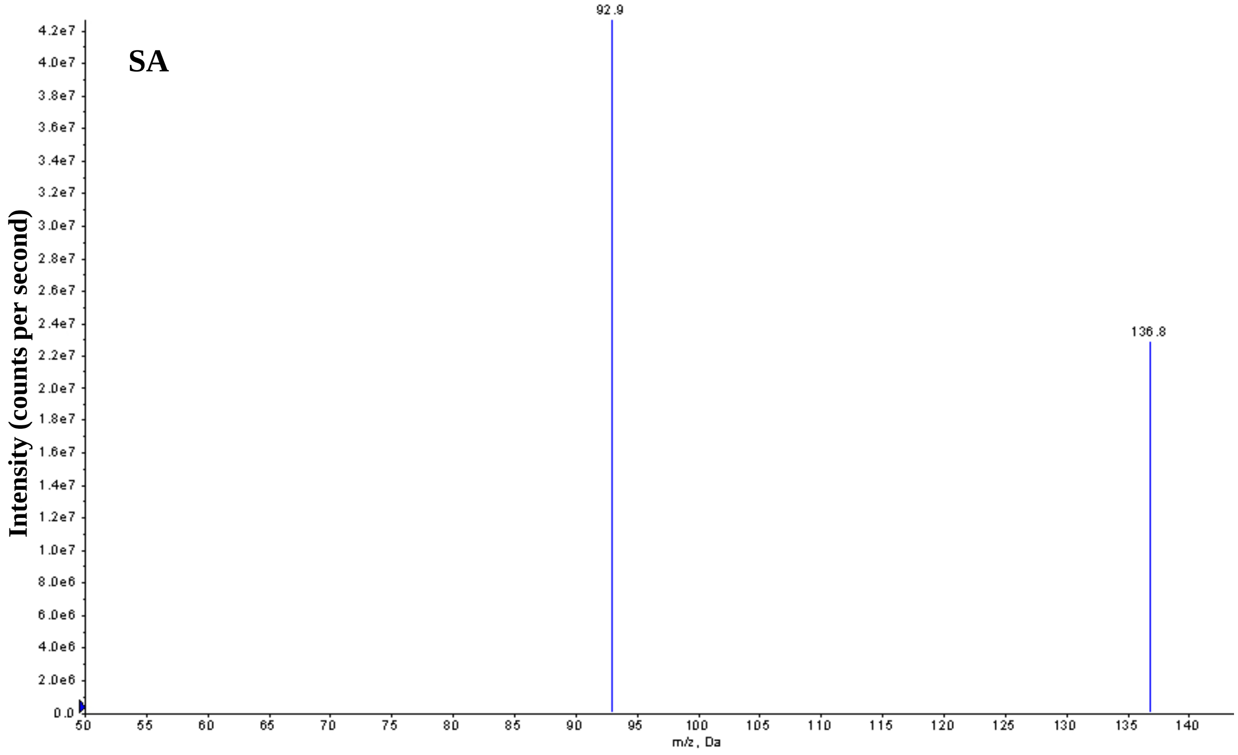

Figure S2 (continued 3). Representative MS fragmentation patterns of analytes.

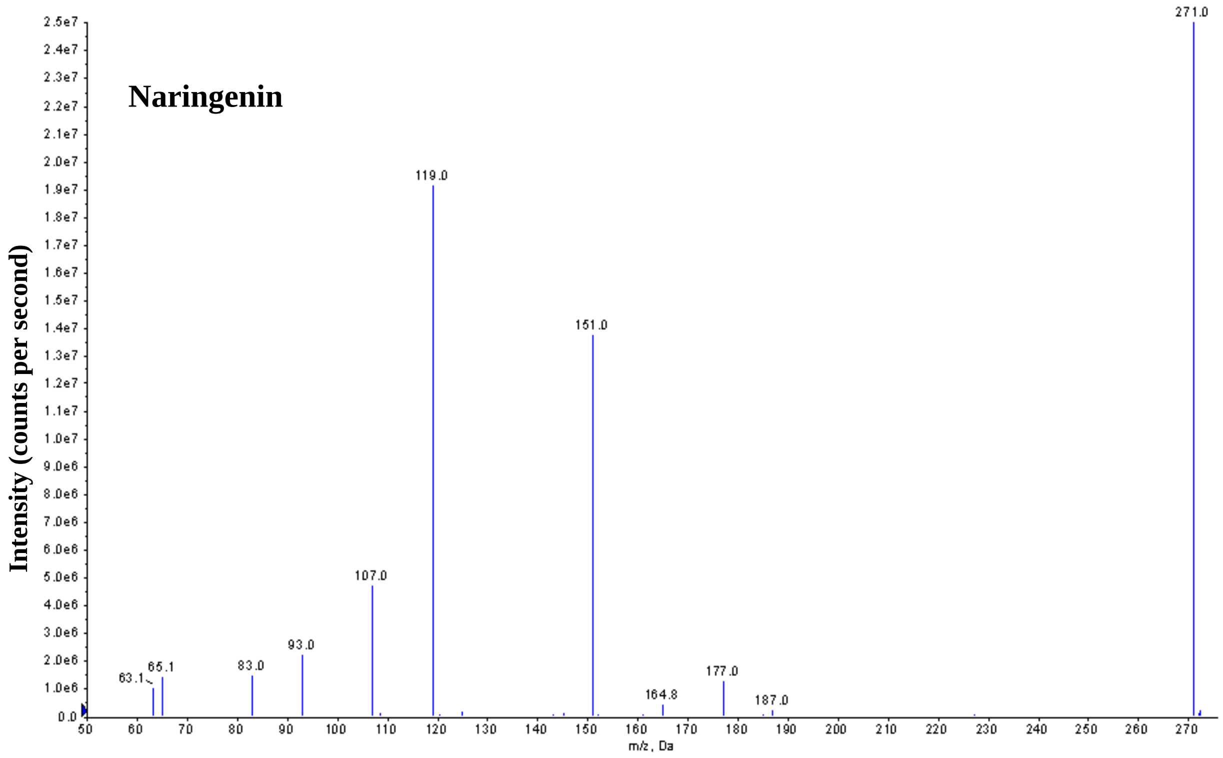

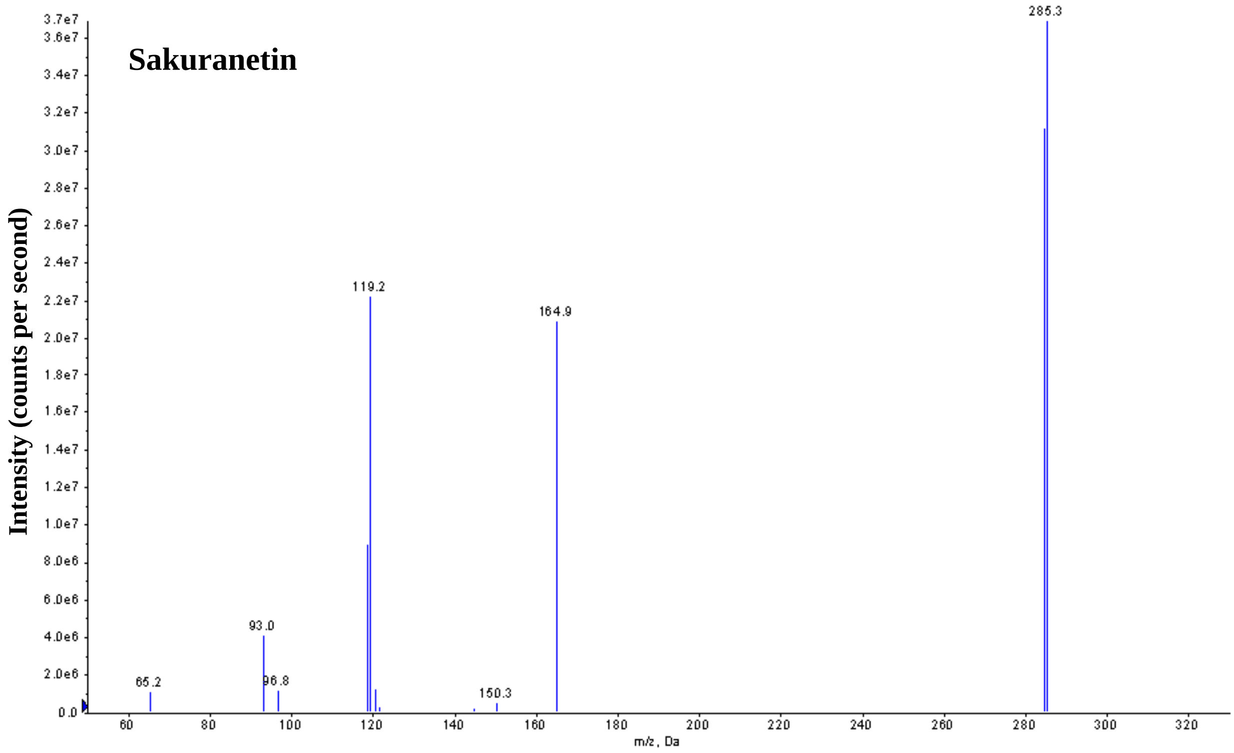

Figure S2 (continued 4). Representative MS fragmentation patterns of analytes.

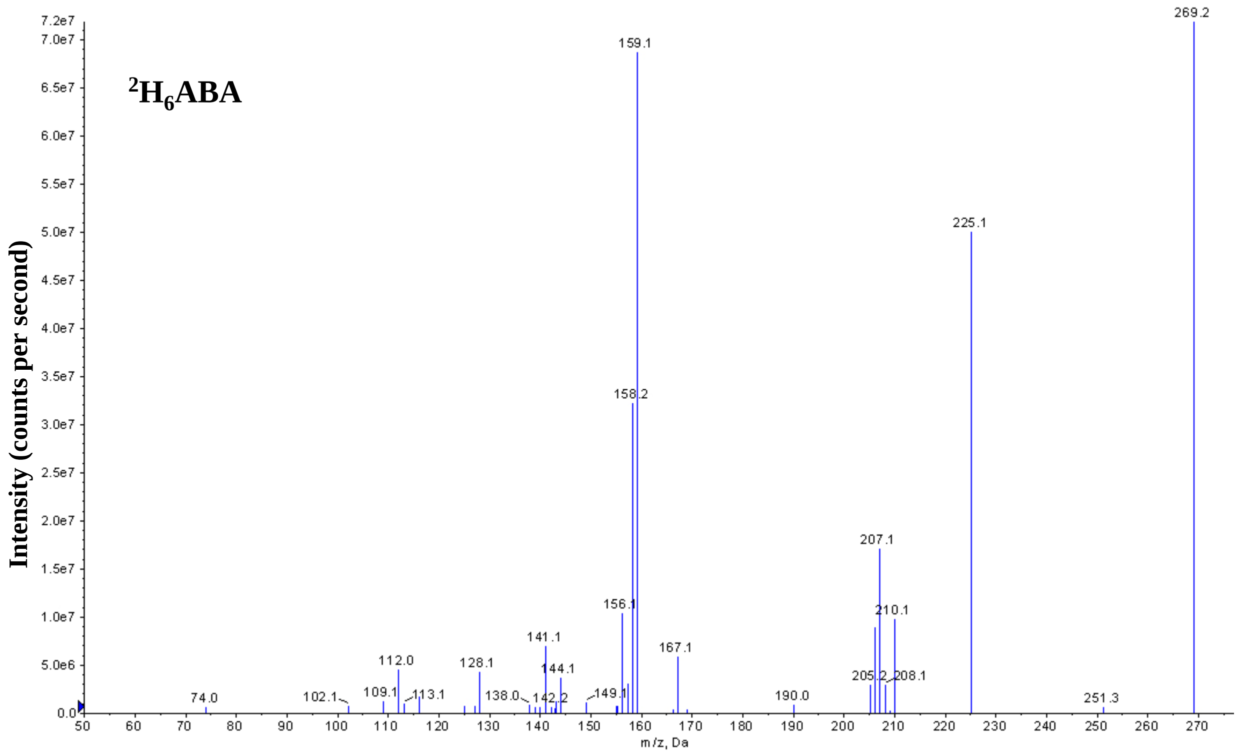

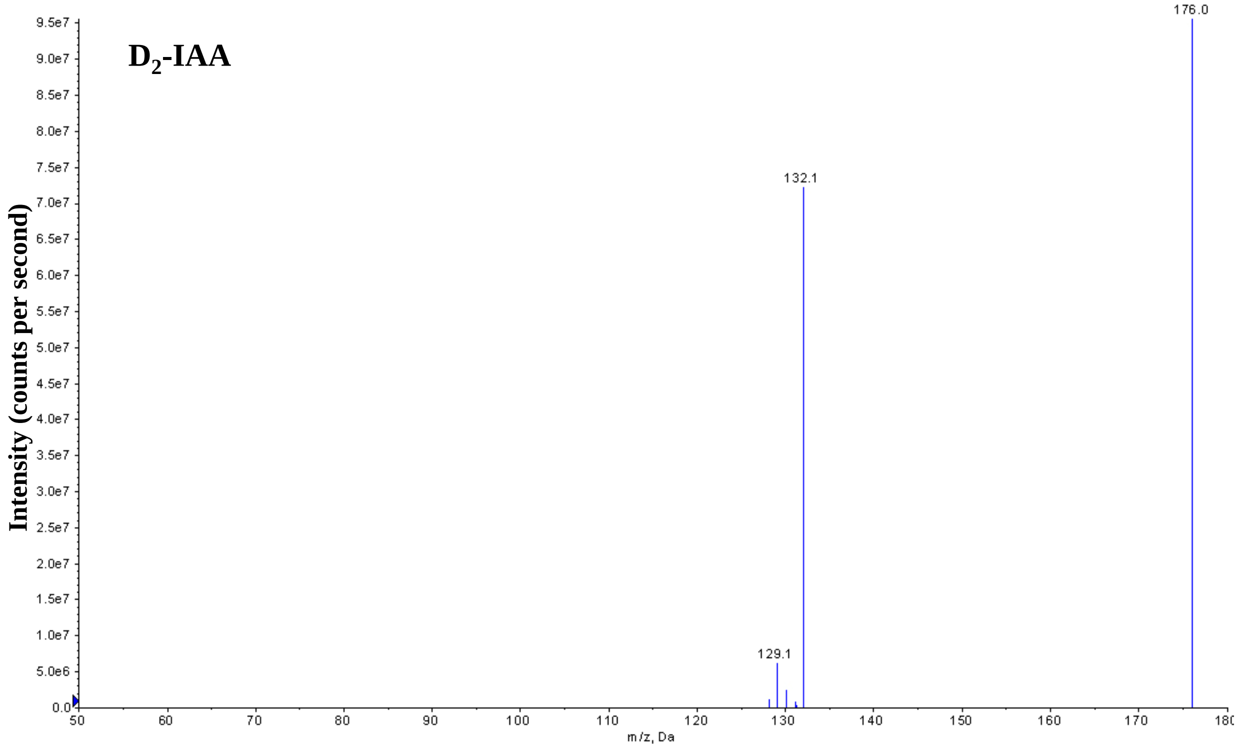

Figure S2 (continued 5). Representative MS fragmentation patterns of analytes.

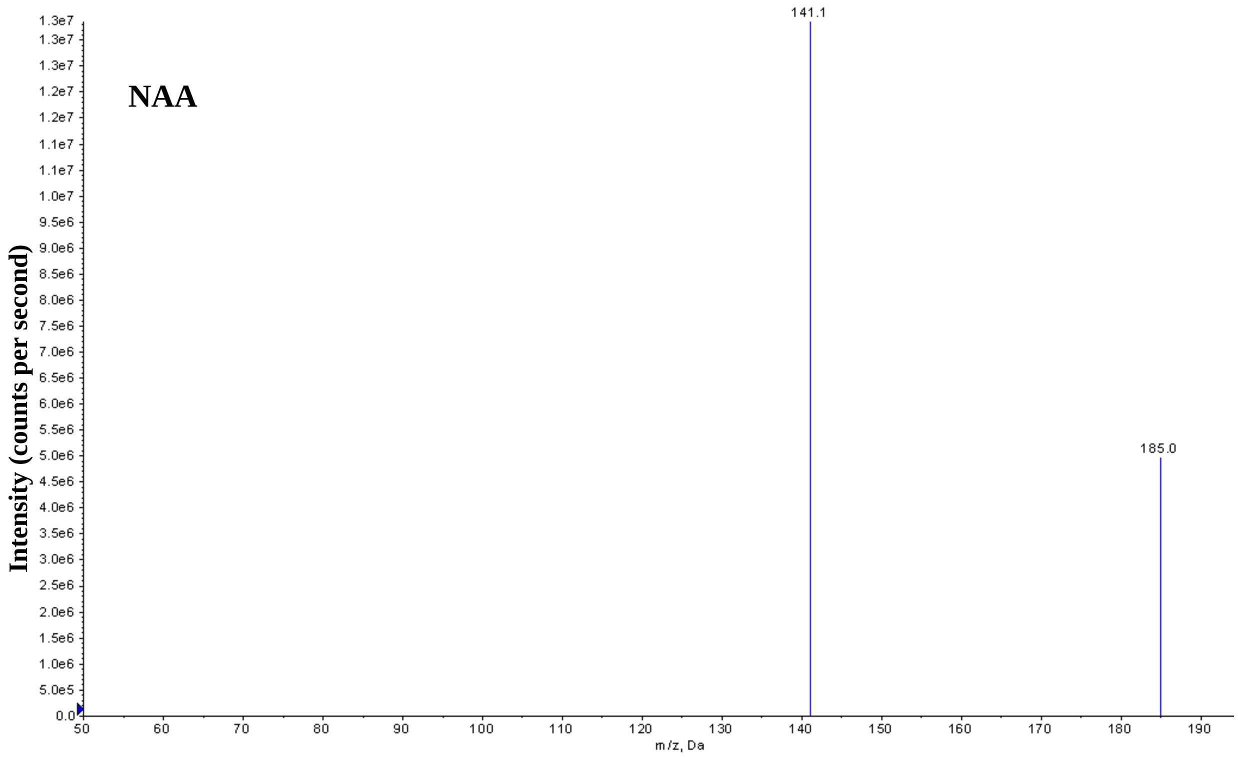

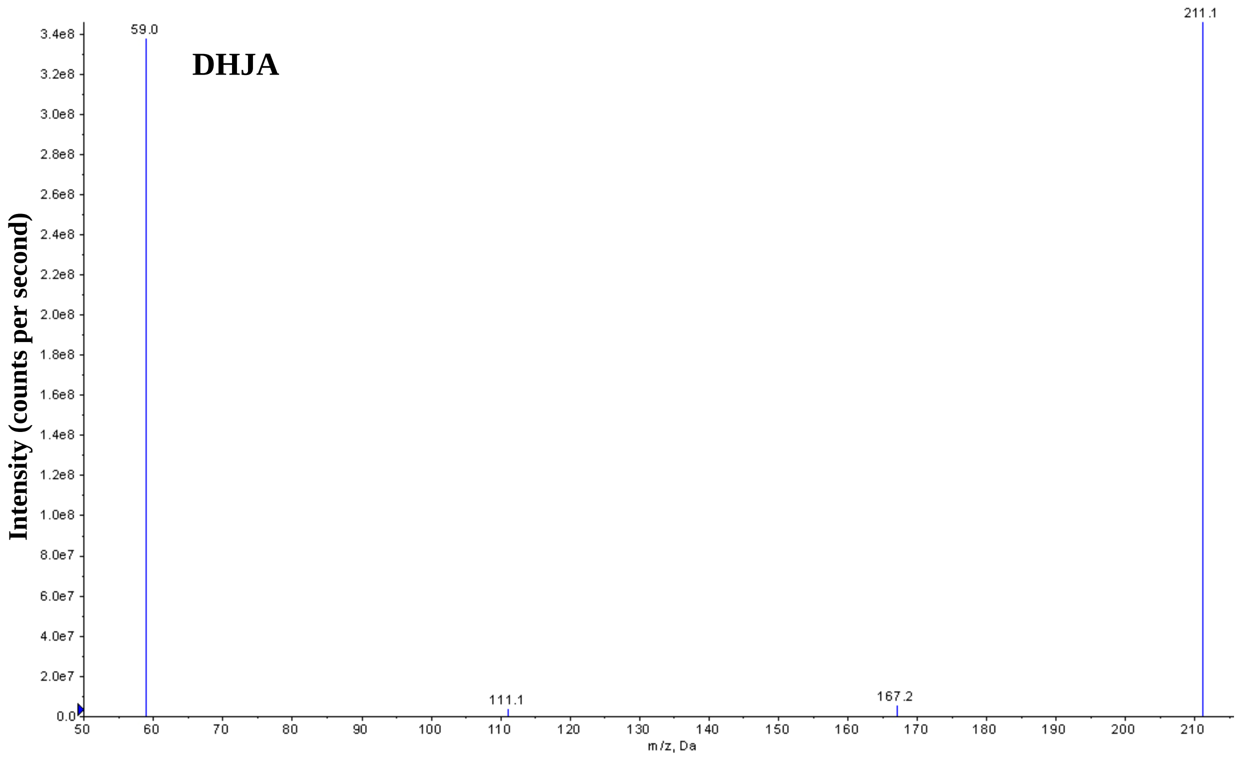

Figure S2 (continued 6). Representative MS fragmentation patterns of analytes.

Table S1. Recovery of analytes in standard-containing samples prepared using different filters and in rice samples

**Analyte**	**Retention time (min)**	**Recovery rate (%)**			**Recovery rate (%)**	
		**Standard after nylon filter-filtration**	**Standard after cellulose filter-filtration**	***P* value[a]**	**Rice sample**	***P* value[b]**
IAA	15.86	92.4 ± 2.1	90.3 ± 6.2	0.7238	89.8 ± 1.3	0.2808
JA	16.95	90.9 ± 1.7	85.9 ± 8.8	0.5741	89.5 ± 1.4	0.4827
JA-Ile	17.38	91.3 ± 3.9	89.6 ± 7.5	0.8033	89.5 ± 1.4	0.4827
ABA	16.32	93.6 ± 1.1	90.6 ± 3.2	0.4259	92.7 ± 0.9	0.4443
SA	8.28	91.2 ± 4.9	92.2 ± 5.8	0.8665	95.2 ± 1.3	0.7577
IAA-Asp	14.27	91.6 ± 1.7	93.7 ± 2.7	0.4626	89.8 ± 1.3	0.2808
MeJA	18.53	90.2 ± 1.0	90.6 ± 0.4	0.6921	89.5 ± 1.4	0.4827
Momilactone A	19.40	92.5 ± 5.6	90.3 ± 2.7	0.7041		
Sakuranetin	17.95	95.3 ± 1.7	90.3 ± 1.1	0.0741		
Naringenin	16.8	94.5 ± 3.1	91.2 ± 0.4	0.3764		

[a]Comparison between standard phytohormones and metabolites after nylon filter-filtration and cellulose filter-filtration.

[b]Comparison between standards after nylon filter-filtration and phytohormones and metabolites in rice samples.

Table S2. Primers used for quantitative reverse-transcription-PCR analysis

Gene (GenBank accession number or RGAP locus number)[a]	Primer name	Forward primer (5'-3')	Reverse primer (5'-3')
ZEP (Os04g37619)	ZEP-F/R	GGATGCCATTGAGTTTGGTT	TGGCTGACTGAAGTCTCTCG
NCED1 (AY838897)	NCED1-F/R	CTCACCATGAAGTCCATGAGGCTT	GTTCTCGTAGTCTTGGTCTTGGCT
NCED3 (AY838899)	NCED3-F/R	CGCAACAGTAAAAAGAATTAACAGC	TATACACACACGCGGTCGTT
NIT1 (AK104033)	NIT1F/R	CACCCCGGCCACTCTAGATA	GATAGCCGCCAACAAAAGCT
AAO3 (AK065990)	AAO3F/R	TTCACCAGCTGAAGGCACAA	GCTCATCCAAGCCATTAGCAA
CHS (X89859)	CHSF/R	CCGGCGAACTGCGTGTAC	TTCCTGATCTGCGACTTGTCA
AOS2 (AY062258)	AOS2-F/R	CAATACGTGTACTGGTCGAATGG	AAGGTGTCGTACCGGAGGAA
ICS1 (AK120689)	ICS1-F/R	TATGGTGCTATCCGCTTCGAT	CGAGAACCGAGCTCTCTTCAA
LOX (D14000)	LOX-F/R	GCATCCCCAACAGCACATC	AATAAAGATTTGGGAGTGACATATTGG
PAL1 (X16099)	PAL1-F/R	GGGCAACCCAGTGACCAA	CGATTGCCTCGTCGGTCTT
KSL4 (AK119327)	KSL4-F/R	CAACAATGGAGGAATACA	AACAACTCATCATACTCTG
CPS4 (AK100631)	CPS4-F/R	TGCCAAGATTCCAAGAGA	GTCCAAGTCAACCATTCC
Actin (X15865)	Actin-F/R	TGTATGCCAGTGGTCGTACCA	CCAGCAAGGTCGAGACGAA

[a]GenBank, http://ncbi.nlm.nih.gov; RGAP, Rice Genome Annotation Project (http://rice.plantbiology.msu.edu).
